# Supplementary material for: Causal interplay between lactose intolerance and gut microbiota: a combined bidirectional Mendelian randomization and in vivo validation study
Source: Front Nutr. 2026 Jun 1;13:1803337. doi: 10.3389/fnut.2026.1803337 (PMC13265576; doi:10.3389/fnut.2026.1803337)
Supplement: Supplementary file 1 [file Data_Sheet_1.zip › supplementary materials/Forward/leave one out cross validation/ebi-a-GCST90027519.finngen_R12_E4_LACTONAS.pdf]

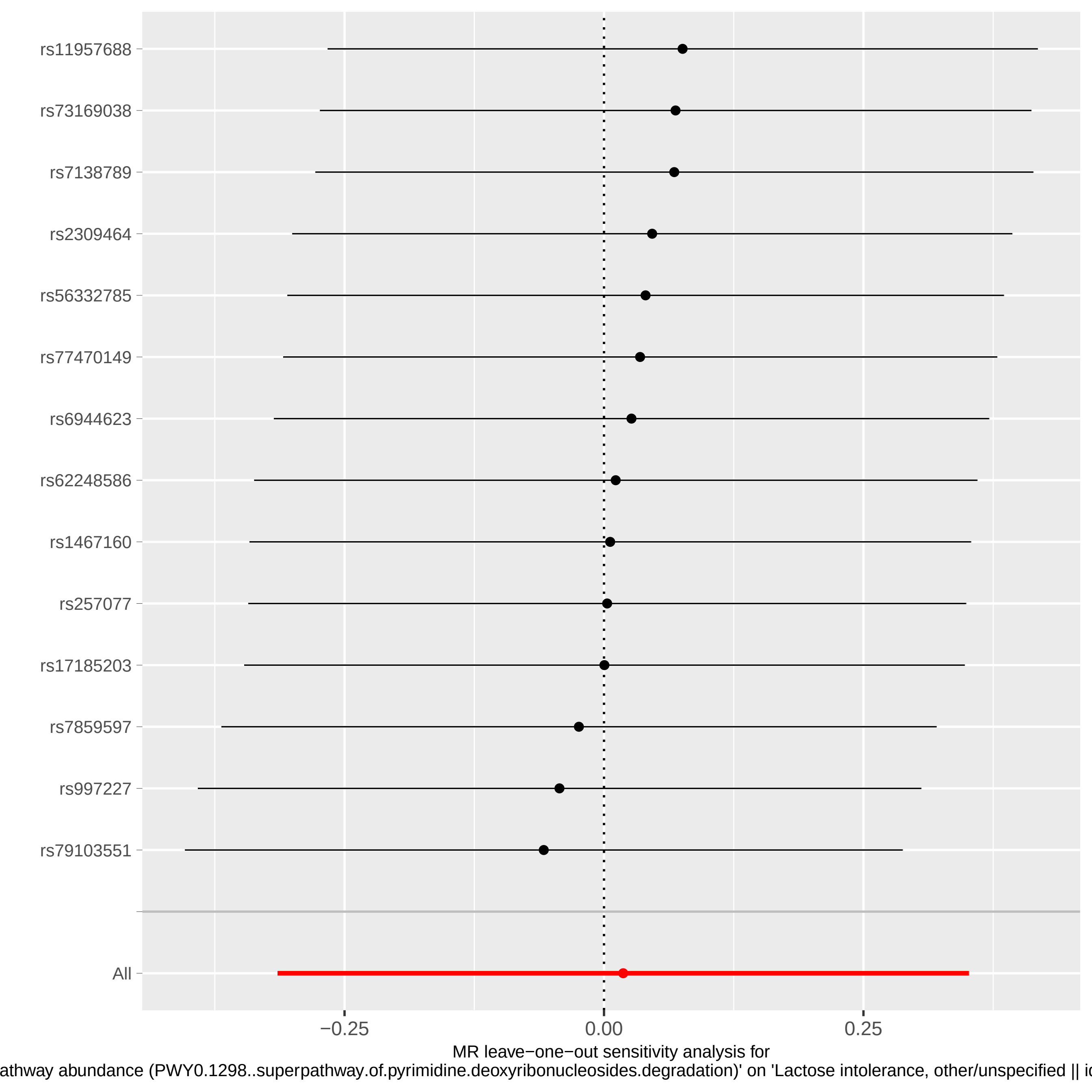
rs11957688
rs73169038
rs7138789
rs2309464
rs56332785
rs77470149
rs6944623
rs62248586
rs1467160
rs257077
rs17185203
rs7859597
rs997227
rs79103551
All
−0.25
0.00
0.25
MR leave−one−out sensitivity analysis for
athway abundance (PWY0.1298..superpathway.of.pyrimidine.deoxyribonucleosides.degradation)' on 'Lactose intolerance, other/unspecified || ic
